# Nutraceutical and Pharmaceutical Behavior of Bioactive Compounds of Miracle Oilseeds: An Overview

**DOI:** 10.3390/foods11131824

**Published:** 2022-06-21

**Authors:** Sonia Morya, Farid Menaa, Cecilia Jiménez-López, Catarina Lourenço-Lopes, Mona Nasser BinMowyna, Ali Alqahtani

**Affiliations:** 1 Department of Food Technology & Nutrition, School of Agriculture, Lovely Professional University (LPU), Punjab 144001, India; 2 Department of Internal Medicine and Nanomedicine, California Innovations Corporation (Fluorotronics-CIC), San Diego 92037, CA, USA; 3 CINBIO, Neurocircuits Group, University of Vigo, Vigo 36310, Spain; cecilia.jimenez.lopez@uvigo.es; 4 Nutrition and Bromatology Group, Analytical and Food Chemistry Department, Faculty of Food Science and Technology, University of Vigo, Vigo 36310, Spain; c.lopes@uvigo.es; 5 College of Applied Medical Sciences, Shaqra University, Shaqra 11961, Saudi Arabia; m.mwena@su.edu.sa; 6 Department of Pharmacology, College of Pharmacy, King Khalid University, Abha 62529, Saudi Arabia; amsfr@kku.edu.sa

**Keywords:** oilseeds, health benefits, micronutrients, bioactive compounds, extraction methods, nutraceuticals, functional foods

## Abstract

India plays an important role in the production of oilseeds, which are mainly cultivated for future extraction of their oil. In addition to the energic and nutritional contribution of these seeds, oilseeds are rich sources of bioactive compounds (e.g., phenolic compounds, proteins, minerals). A regular and moderate dietary supplementation of oilseeds promotes health, prevents the appearance of certain diseases (e.g., cardiovascular diseases (CVDs), cancers) and delays the aging process. Due to their relevant content in nutraceutical molecules, oilseeds and some of their associated processing wastes have raised interest in food and pharmaceutical industries searching for innovative products whose application provides health benefits to consumers. Furthermore, a circular economy approach could be considered regarding the re-use of oilseeds’ processing waste. The present article highlights the different oilseed types, the oilseeds-derived bioactive compounds as well as the health benefits associated with their consumption. In addition, the different types of extractive techniques that can be used to obtain vegetable oils rich from oilseeds, such as microwave-assisted extraction (MAE), ultrasonic-assisted extraction (UAE) and supercritical fluid extraction (SFE), are reported. We conclude that the development and improvement of oilseed markets and their byproducts could offer even more health benefits in the future, when added to other foods.

## 1. Introduction

**Figure 1:**
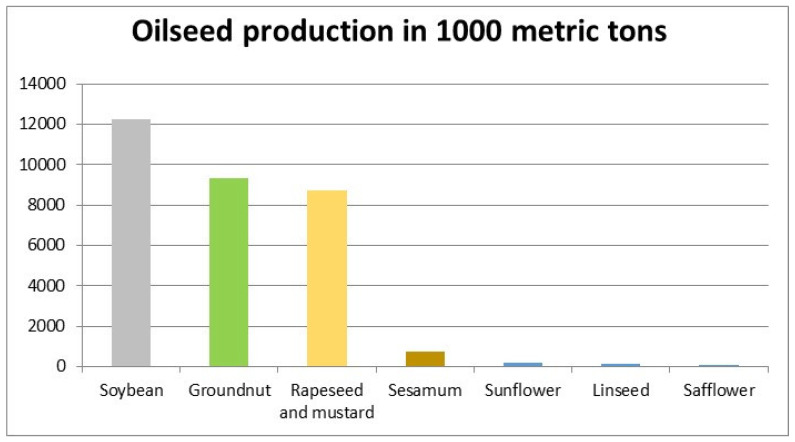
Production scenario of different oilseeds in India in 2020 (adapted from Statista Research Department, https://fr.statista.com/a-propos/notre-engagement-pour-la-recherche (accessed on 2 November 2020).

**Figure 2:**
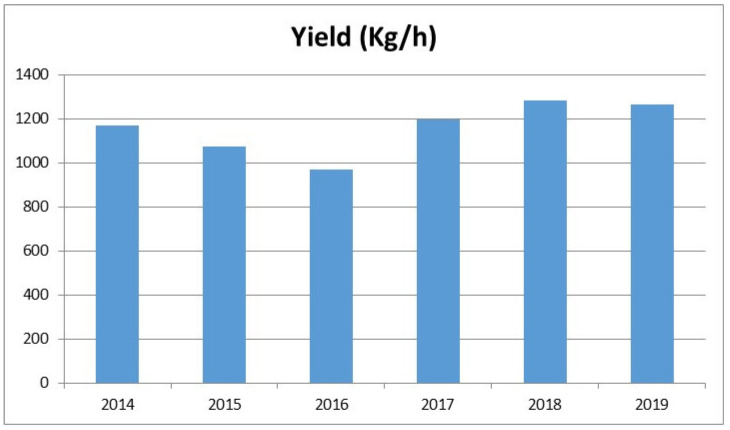
Annual yield (per hectare) of oilseeds in India (adapted from Statista Research Department, https://fr.statista.com/a-propos/notre-engagement-pour-la-recherche, Accessed on 16 October 2020).

Vegetable oils are extracted from plant seeds (oilseeds) and are mainly used in the food industry (e.g., preparation of nutritional food products, healthy snacks). In 2003/2004, 39.8 million oilseed stocks have been estimated around the world [1], with India being one of the major producers of the main oilseed crops, and one of the highest consumers of vegetable oils in the world (Table 1, Figure 1 and Figure 2). The principal oilseed crops in India are groundnut, rapeseed, mustard, castor, sesame, linseed, sunflower, soybean, sunflower, and coconut. These oils are abundant in essential nutrients, such as *n*-3 and *n*-6 unsaturated fatty acids, peptides and proteins, vitamins, and different bioactive compounds (e.g., tocopherols, phenolic compounds, phytosterols) [2]. Therefore, oilseeds are quite valuable in terms of their nutrient content and beneficial properties (Table 2).

Proteins are essential for the proper functioning of the human body, acting as enzymes, carriers, hormones or antibodies, repairing and building tissues, and carrying out structural support. Thus, protein content in food systems is significant due to its functional properties [3], and to the changes in dietary trends in society, which require alternative sources of protein. Soybeans, cottonseed, rapeseed, peanut, and sunflower seeds are rich in protein and contribute to the world production of protein meal at 69%, 6.9%, 12.4%, 2.8% and 5.3%, respectively. Additionally, proteins contained in oilseeds generally present well-balanced profile [4].

Regarding lipids and in general terms, vegetable oils show higher amounts of unsaturated fatty acids (UFAs) which reduce the risk of CVDs when compared to solid animal fats, which present higher amounts of saturated fatty acids (SFAs) and trans-fatty acids (TFAs). SFAs and TFAs consumption increases low-density lipoprotein (LDL) content in the body, which raises the risk of CVDs. Hence the inclusion of vegetable oils in the diet promotes human health [5,6].

It should be noted that there are also naturally occurring TFAs in dairy and certain meat products. On average they represent only 2–6% of fatty acid composition in dairy products and 3–9% of the fats in cuts of beef and lamb [6]. In contrast to industrial TFAs, a moderate intake of natural TFAs does not appear to be harmful [6,7,8,9].

Fats and oils represent the most concentrated energy sources, providing approximately 9 Kcal per gram of energy compared to proteins and carbohydrates, i.e., about 4 kcal per gram [10]. The increased demand for human consumption as well as the industrial use of vegetable oils has encouraged the growth and efficient production of pure and high-quality oils [11,12]. However, industrial extraction techniques and/or further thermal treatments carried out during the production of oils may compromise the amino acid, lipidic, and other beneficial molecules’ content [13].

The aim of this review is to highlight the different types of oilseeds, their derived bioactives and beneficial human health effects.

## 2. Extraction Methods for Oilseeds

From the beginning of human civilization, traditional methods have been used by rural communities to extract edible oils from plant-based materials (e.g., peanut, corn, coconut, soybean, palm), due to their high nutritive value [15]. Those methods have evolved over a long time and new techniques have come out trying to maximize the efficiency of the process. Nevertheless, oilseeds extraction yield will depend on the variety of oilseeds, the oil-bearing plant’s environmental and growing conditions, and on the specific extraction method and pretreatment procedures employed. The principal extractive technique to obtain vegetable oil from oleaginous materials is the Soxhlet-based solvent extraction process. However, this method is commonly used for oil extraction just on a laboratory scale [16], while at an industrial scale, solvent extraction is mostly used because of its easy scalability [17].

Extractive techniques are divided into two major classes; however, they are frequently combined to profit from the benefits of each one. It is usual to find protocols in which an innovative methodology (e.g., microwave pretreatment) is performed prior to applying a conventional technique (e.g., mechanical extraction) to maximize the results.

### 2.1. Pretreatment of Oilseeds

Numerous extraction methods require an oilseed’s’ pretreatment. Seed coat elimination, cleaning, sorting, dehulling, grinding, winnowing and preheating are the basic steps employed during this process [18]. For instance, heating treatment further helps in the process of oil release by decreasing the moisture content and hardening the seed oil interior [11]. However, other factors such as materials’ moisture content, temperature and particle size can also be controlled during pretreatment to increase the final yield of the extracted oil. The processing or pretreatment of oilseeds offers a means of controlling the main parameters and conditions to improve oil quality and oil yield [19]. Roasting seeds is one of the most used pretreatments and can be performed in an oven or by dry air. Of course, the conditions selected for the process will affect the final oil composition [20].

Other more elaborated pretreatments require seeds’ cell membranes alteration, which improves the compounds’ flow and further extraction. Those methods involve the use of microwaves, ultrasounds, electric pulses, fungal pretreatment, enzymatic hydrolysis, acidic hydrolysis [21,22,23], or steam explosion, the last one combining a temperature of 160–260 °C with a pressure of 0.69–4.83 MPa for some seconds to a few minutes, which favors oil and bioactive compounds release, and increases extraction yields up to 7.4% [24].

### 2.2. Traditional Extractive Techniques

The old traditional ways of oil extraction from seeds involved the roasting of kernel seeds by mortar and pestle, the blending of the crushed material with water, the cooking of the blended paste to attain the oil by floating and skimming, and the drying of oil by heating [25]. However, these methods are time-consuming, tedious, and lead to low yield and quality. Nowadays, two traditional methods of oil extraction from oleaginous plant materials can be highlighted: solvent and mechanical methodologies [11].

#### 2.2.1. Solvent Extraction

The extraction of an oil can be performed using a solvent to remove the oil from the vegetable matrix, by coming in direct contact with it and sometimes using heat and agitation. Based on this principle, there are several different methodologies that can be used (e.g., maceration, Soxhlet extraction, infusion, distillation). Solvent extractions are conventional methods applied to oilseeds containing low oil contents (<20%), such as soybean. These procedures are known to be the most effective ways of extracting vegetable oils, with a reduced quantity of remaining oil lasting in the meal or cake [26]. The solvent selection is primarily based on the greatest leaching properties of the required solute substrate [27]. Ethanol, diethyl ether, hexane, and petroleum ether are the solvents commonly used for solvent extraction methods and provide a variety of benefits. In a study conducted by [28], which consisted of enhancing process parameters for the production of castor oil, the authors discovered that solvent oil extraction capability during solvent extraction is improved with an increase in the time of extraction. Besides, ref. [29] observed the impact of extraction techniques on the quality and yield aspects of oils from shea nut. They compared the outcomes of oils’ chemical, physical and sensory properties, when extracted using a traditional technique versus a solvent extraction process. For the solvent extraction process, they showed a higher oil yield of 47.5% compared to 34.1% for the traditional process, and good long-term stability for the solvent extracted oil.

However, these techniques have many industrial drawbacks. For instance, a high amount of solvent consumption, high energy requirements, emissions of volatile organic compounds into the atmosphere, low quality of the product due to the high temperature applied during the process, long extraction hours, high monetary investment, many processing, and problems with plant protection [30]. Besides, due to the use of organic solvents, these methods require extra expenses and more labor time for their complete removal [31].

Soxhlet extraction is one of the main ways to remove vegetable oils from the oleaginous materials. On a laboratory scale, the Soxhlet extraction method is commonly used [16], but for large scale operations, this method needs a commercial extractor for the solvent. The great benefit of this technique is the recycling of the organic solvent during the extraction. Nevertheless, the solvent demand is still too high and is a time and energy consuming process [32].

#### 2.2.2. Mechanical Extraction

Mechanical extraction includes the use of pressure to extract oil from an oil containing material. The pressure is applied by using screw pressure or hydraulic forces. By these processes, an increase in mechanical pressure on the oil-bearing material promotes the extraction of the oil and increases the yield. In normal operating conditions, screw pressure extraction usually allows the obtention of bigger yields than hydraulic extraction [33]. Mechanical extractions are commonly used to produce vegetable oil from oilseeds with an oil content higher than 20% [34].

This technique can be performed according to temperature, resulting in cold and hot-pressed oils. The process for production of cold-press oils is conducted at low temperature and pressure (normally, under 5 °C), while in a hot-press process, high temperature and pressure are applied. Cold-pressing is achieved using hydraulic or screw press coupled to a cooling system, and the obtained oils are healthier than hot-pressed seed oils because high temperatures usually lead to degradation of bioactive and functional ingredients, with repercussions on the quality of the oil obtained. In the cold pressed oils, the purity and natural characteristics of seed oils are maintained, and higher phenolic compounds, tocopherols, phytosterols, fatty acids and carotenoids contents are found. This technique is the most employed to extract oil from rapeseeds, after proper seed pretreatment [19,35,36]. Due to their attractive characteristics and health benefits, there is a rising world market for cold-pressed oils. Nevertheless, the hot-pressed technique produces bigger oil quantities because the temperature applied reduces the viscosity of the oils in the seeds, which increases the flow of oil during extraction. Thus, higher temperatures improve the effectiveness of the extraction and yields up to 80% can be attained. However, as said before, the oils obtained by this process are poorer in bioactive compounds and, thus, have less health benefit [11].

When comparing with solvent extractions, mechanical extractions present several advantages. At a small scale, this methodology is easy, safe and involves less steps than the solvent alternative [37]. Furthermore, it does not require the use of organic solvents (i.e., eco-friendly practice) and allows the obtention of bigger extraction yields because the oil is almost totally removed from the remaining meal or cake [19,38].

### 2.3. Innovative Extraction Techniques

#### 2.3.1. Microwave-Assisted Extraction (MAE)

MAE is an advanced method for the isolation of vegetable oils from oilseeds, based on the application of radiation to produce the rotation of water molecules present in the plant matrixes. This approach is gaining interest, as microwaves produce holes in cellular membranes, favoring their permeability and increasing oil extraction yields. Indeed, essential oils can also be extracted with this method [19,32]. It has been reported that oils obtained from rapeseeds through MAE exhibit a significantly better oxidative stability, most probably because of the improvement in phenolic antioxidant compounds. A study reported that pretreated oils extracted using MAE show a greater concentration of UFAs and essential FAs, and therefore a subsequent improved oil quality [39]. MAE has been used for oil extraction from different oilseeds such as hazelnuts, olives, peanuts, soybeans, rapeseeds, sunflowers, canola, and castor [40]. The benefits of this method are improved oil extraction and production, direct extraction, reduced power usage, and a fast processing period (in the order of minutes in some cases) [35].

The microwave technique is often applied as a pretreatment, preceding other extractive techniques. Cracking cell membranes leads to higher extractive efficiency. For instance, the microwave pretreatment of rapeseeds before the cold-pressing method showed increases in extraction yield of up to 64%, while conserving bioactive compounds such as phytosterols, tocopherols, and phenolics, and improving the oil oxidative stability [19,41]. Furthermore, microwave pretreatment has been reported to enhance phenolic compounds’ preservation, such as canolol, during oils storage at room temperature, extending their shelf life by up to 12 months [42].

#### 2.3.2. Ultrasonic-Assisted Extraction (UAE)

UAE is a modern and creative technology that uses ultrasound waves for increasing heat and molecular vibration. Thereby, UAE includes high-frequency sound waves, of a high-intensity, and their material interaction. It can be used on both a large and small scale [43]. UAE was also used for rapeseeds, soybeans [44], and *Monopterus albus* oil extraction [45]. Li et al. have extracted soybean oil production using hexane as a solvent in the UAE process [44]. The key advantage of UAE is the shorter time of preparation and reaction, the use of limited materials, and minimum spending on solvents. It is very useful for bioactive principles’ purification and isolation [46].

#### 2.3.3. Supercritical Fluid Extraction (SFE)

SFE represents an alternative method of analytical-scale seed oil determination. SFE is used for the extraction from plant materials such as aroma compounds, caffeine, and essential oils. SFE can be modified according to several factors, such as extraction time, sample volume, temperature, co-solvent flow, and pressure. Summarized benefits of SFE over conventional methods include low temperature separation of materials which eliminates thermal damage, no residual solvents which is eco-friendly, improved diffusivity, low viscosity which permits more selectivity, and reduced extraction times [47,48]. In comparison with traditional techniques, SFE is a promising alternative whose optimization for each oilseed can achieve both, an oil yield comparable to the conventional organic solvent extraction, and a product quality similar to mechanical pressing procedures [49].

Besides, this technique is more efficient in the recovery of valuable products, namely tocopherols and phytosterols, from oil wastes than Soxhlet extraction, minimizing toxic organic solvents’ usage, maximizing extracted products’ purity, and contributing to a green circular economy [50].

### 2.4. Limitations of Oilseed Extractions

Although oilseeds contain numerous functional compounds, they also show molecules that can cause negative effects when consumed. That is the case of erucic acid, a toxic MUFA whose intake is restricted, and glucosinolates, which are not suitable molecules for the livestock feed [19]. Pretreating seeds is a valuable approach to reduce those prejudicial compounds’ extraction, while enhancing target compounds’ recovery. For instance, oilseed roasting at 150 °C prior to mechanical extraction has been proven to diminish the glucosinolates content by ≈ 30%, but to increase the total FA content by ≈ 2%, which improves the oil quality [51]. Another study shows that microwave pretreatment (800 W for 5 min) avoids the benzo[a]pyrene accumulation related to oven roasting during peanut butter preparation [52].

Furthermore, to get stable, clear, odorless, pale, bland-tasting oils, the edible crude oil undergoes degummed, deacidified, decolorized and deodorized processes. During such processes, over 95% of the phosphatides, free FAs, carotenoids, glycosides, and approximately 32–61% of the sterols, tocotrienols, and tocopherols, are eliminated. Then, these processes influence the taste, appearance, smell, or storage strength. Vitamin K properties are also diminished during the refining process. This causes the loss of many beneficial compounds from oilseeds to be separated from the final product in the refining process, making its consumption less beneficial, which is why it is recommended to consume unrefined oils whenever possible [50].

## 3. Types of Oilseeds

### 3.1. Peanuts (Arachis hypogae *L.*)

Peanuts, also called monkey nut or goober, are mainly cultivated for oil production and as a food item. It is used more than the third portion all over the world from cultivated peanuts [53]. Peanuts are an invaluable source of calories, proteins, and other micronutrients (e.g., vitamins, minerals, and essential FAs) [54]. Peanuts’ consumption of is associated with health improvements in people suffering micronutrient and energy deficiencies. Besides, enough consumption of peanuts promotes a healthy growth and plays a crucial role in disease prevention. Peanuts provide 534 kcals of energy per 100 g (Table 2) and are also rich sources of minerals such as potassium and phosphorus (Table 3). In addition, peanuts contain up to 30% of protein in their dry weight. Furthermore, these nuts offer a cheap source of high-quality oil and dietary vegetable proteins [55,56,57].

### 3.2. Flaxseed (Linum usitatissimum)

Flaxseed is one of the oldest cultivated crops. It is cultivated widely for its fiber, oil, and as a food product [58]. Flaxseed is consumed worldwide as a cereal, and it is now typically eaten as ground flaxseed meal, flour, or even as whole seeds in North America [59]. It has been suggested that one to three tablespoons of ground flaxseed per day (8–24/day) favors a healthy and balanced diet [59,60]. Flaxseed is also used as animal feed to enhance the reproductive efficiency and well-being of animals [61]. It is one the best sources of omega-3 FAs, being rich in alpha-linolenic acid. It provides 534 Kcal of energy, 18 g of protein, and 27 g of dietary fiber per 100 g of flaxseed (Table 1). The fiber portion is composed of two thirds of insoluble fibers (mostly lignin, cellulose and hemicellulose) and one third of soluble fibers that were shown to decrease cholesterols and help to control blood glucose in the form of mucilaginous material made from polysaccharides [62]. As shown in Table 3, it also contains minerals.

### 3.3. Rapeseed/Canola Oil (Brassica napus subsp. Napus)

Oilseeds of *Brassica napus*, also known as rapeseeds, show a high content of oil, being excellent sources for vegetable edible oils production, or even biodiesel from feed stock oils. For instance, soybeans’ oil content is 20%, which is quite inferior when compared to rapeseeds that contain up to 42% of oil in their seeds [63].

Rapeseed oil is one of the healthiest vegetable oils and, therefore, rapeseed cultivation has raised interest [64]. Essential compounds are present in rapeseed oil, such as sterols, tocopherols, polyphenols, phospholipids (PLs) and flavonoids, which are beneficial when added to the human diet. In addition, rapeseed oil shows remarkable quantities of omega-6 and omega-3 FAs [65]. Originally, rapeseed contained a small amount of erucic acid, but lately new varieties have replaced erucic acid with oleic acid. In the USA, these new varieties are called as canola and result in canola oil [19]. Rapeseed oil provides 884 Kcals of energy (Table 2), which is a remarkably high amount compared to other vegetable oils. It is also abundant in minerals such as calcium, phosphorous, and potassium (Table 3).

### 3.4. Sunflower Seed (Helianthus annuus)

Sunflower is an oilseed crop indigenous to North America. It is grown all over the world, and most of its products are marketed for culinary purposes or livestock feed [66]. The evolution of sunflower to various climatic and soil conditions has increased its cultivation throughout the world as an oilseed plant [67]. This oil can be found as a high-quality edible oil, with culinary applications and several benefits [68]. Additionally, sunflower meal contains minerals, B complex vitamins, essential amino acids, and also has high antioxidant properties, which makes it interesting as a food ingredient for humans and livestock [69]. It is possible to process sunflower seeds into various types including baked, roasted, flour or boiled as composite functional foods [70]. Compared to warmer climates, cooler weather conditions produce higher concentrations of the linoleic acid *n*-6 PUFA, while oleic is the most commonly found MUFA [71]. Sunflower provides 163 Kcals of energy, 21 g of protein, 8.6 g of dietary fiber, and 47.5 g of fat (Table 2), among other micro and macro nutrients (Table 3).

### 3.5. Sesame Seeds (Sesamum indicum)

Sesame (red, black, or white) is known to be one of the oldest cultivated crops and oilseed plants with many benefits to human health. It is present in the tropics and subtropics, and is most widespread in the narrower belt near to the Equator [72]. India is one of the leading producers of sesame seeds in the world. Sesame is also popularly known as the “Queen of Oilseeds” because of its higher resistance to rancidity and oxidation [73]. Sesame seeds are a rich source of phytosterols, sulfur, and amino acids [74]. Some of the antioxidants present in this oil are polyphenols sesamol, lignans, sesamolin, and sesamin. The sesaminol glucosides and tocopherol, also present in this oil, keep the oil stable and contribute to a longer shelf-life compared to that of other vegetable oils [75]. Sesamolin and sesamin, related to lignans, a class of beneficial fibers, elicit positive effects on human health by lowering cholesterol, preventing elevated blood pressure, and in animals by increasing vitamin E blood levels [76]. Sesame is also useful as a protein source (i.e., presence of methionine balanced amino acids), especially for Kwashiorkor patients. Due to nutritious protein and good quality oil, sesame has a high food value. It provides 573 Kcals of energy, 18 g of protein and 12 g of dietary fiber (Table 2). Besides, it is also a good source of calcium, phosphorus, potassium, and magnesium (Table 3).

### 3.6. Soybean (Glycine max)

Soybeans are a rich nutritious crop and play a major role in solving nutritional deficiency and food scarcity problems, mainly in developing countries. Thereby, the intake of soybean related foods has a long tradition in African and Asian countries. Soybeans and soya products have played a significant role in Asian cuisine for many centuries. Traditional soy foods such as tofu, tempeh, miso, and soya sauce are extracted either from whole fresh beans or beans processed into soymilk. Increased soybean demand has emerged due to growing soybean oil and soybean milk consumption and its demand continues to grow.

Soybeans provide 173 Kcal, 7.3 g of fat, 36 g of protein per 100 g of seeds (Table 2), as well as 83 mg of calcium and 3 mg of iron per 100 g of seeds (Table 3).

### 3.7. Cottonseed (Gossypium)

Nowadays, cottonseed oil supplies 4% of the world’s vegetable oil production; even though, its growth is associated to cotton fiber demand. This vegetable oil provides 367 Kcals of energy, 49 g of protein and 5.5 g of dietary fiber per 100 g of oil (Table 2). Potassium levels are remarkably high in cottonseed (Table 3). The enhanced oxidative stability of cottonseed oil is provided by its high content in oleic acid which may also have advantages in industrial-scale frying, due to the decreased oil use and extended shelf-life of the products [77].

### 3.8. Safflower (Carthamus tinctorius)

Safflower is one of the valuable oilseeds crops that is used for the confection of protein cakes. It is grown in South America, North America and Asia [78]. Safflower is primarily cultivated for vegetable oil production from its seeds. The conjugated linoleic acid present in safflower oil was found to reduce body weight and adipose tissue in some clinical trials [79], and seems to be effective in fat-induced insulin resistance [80]. Safflower seeds and oil inhibit degenerative diseases that affect the health of the bones and joints. A few studies recently planned to use safflower seeds and oil to produce functional foods [81,82]. Additionally, traditional and oriental medicines are known to contain safflower seeds to cure chicken pox sores, neuropathy, tingling and numbness. Safflower oil is used in India, Japan and the USA to regulate blood parameters such as high density lipoprotein (HDL) and cholesterol [82]. This oil provides 517 Kcals of energy, 16 g of protein per 100 g of seeds (Table 2) and contains a high amount of potassium and phosphorus (Table 3).

## 4. Nutraceutical and Pharmaceutical Aspects of Bioactive Compounds Present in Oilseeds

Oilseeds are a great source of affordable and potent bioactive compounds (e.g., carotenes, flavonoids, PUFAs, organosulphur compounds, phytosterols, and polyphenols) generally used in the pharmaceutical (anti-microbial toxins, adjuvants for cancer therapy, cholesterol-lowering therapeutics), agricultural (animal welfare) and cosmetic (oil-based creams) industries. However, oilseeds also contain certain molecules called anti-nutrients, which are toxic compounds synthesized by plants as a defense mechanism. Controlling these compound’s amounts, namely glucosinolates and phytic acid, is essential to maximize beneficial effects over toxicity [83,84].

### 4.1. Glucosinolates

Glucosinolates are secondary metabolites of sulfur-rich anionic plants [85,86]. Their structure comprises of a moiety of β-D glucose bound to sulphatated thiohydroximate. About 100 different glucosinolates were discovered from 16 families of plants, namely *Bataceae*, *Bretschneideraceae*, *Phytolaccaceae*, *Resedaceae*, *Moringaceae*, *Tovariaceae*, *Tropaeolaceae*, *Limnanthaceae*, *Gyrotemonaceae*, *Euphorbiaceae*, *Cruciferae* (syn. *Brassicaceae*), *Pentadiplandraceae*, *Pittosporaceae*, *Capparaceae*, *Caricaceae*, and *Salvadoraceae* [87,88].

Glucosinolates are present predominantly in Brassicaceae vegetables, which include mustard, rapeseed, broccoli, cauliflower, cabbage, and brussels sprouts, among others [89]. Earlier reports revealed biocidal, anti-oxidant, bioherbicidal and antineoplastic properties of glucosinolates and derived by-products present in Brassicaceae vegetables [90,91]. They express tolerance/resistance to pests and diseases and gathered in the organs leading to tolerance/resistance at a relevant development stage [92,93]. It has been proven that glucosinolates and their derivatives have beneficial effects on human health by minimizing the danger of some cancers [94]. Epidemiological research has linked consumption of Brassicaceae vegetables with a decrease in cancer incidence (including stomach, rectum, lung and colon cancers) (Table 4). Dietary glucosinolates were found to block the emergence of exogenous or endogenous carcinogens to avoid the activation of carcinogenesis [91]. These healthy properties are mainly attributed to their hydrolytic products derived from the metabolism: isothiocyanates. In contrast, glucosinolates produce a toxic effect when ingested by livestock, in particular, pigs seem to be more affected by dietary glucosinolates than ruminants, rabbits, poultries, or fish. Glucosinolates’ toxicity includes enlarged thyroid and reduced thyroid hormone levels; liver, kidney and growth abnormalities; reduced reproduction rate, and even mortality [88].

### 4.2. Phenolic Compounds

Phenolic compounds are other secondary metabolites of plants whose original function is mostly defensive. Although they can be found in all plant parts, phenolic composition is really variable between them and between plants, both, quantitatively and quantitatively [122]. Phenolic compounds comprise a broad group of molecules, that can be classified into five major categories: phenolic acids (hydroxycinnamic acids and hydroxybenzioic acids); stilbenes; quinones; flavonoids (anthocyanins and anthocyanidins, flavonols, flavones and isoflavones); and tannins and lignans. In food matrixes, they are usually related to the organoleptic properties (color and taste), although they also perform preservative functions, and have beneficial effects for human health [123]. These beneficial properties are mainly attributed to their remarkable antioxidant capacity, which participates in the prevention of different illnesses linked to oxidative stress such as premature aging, neurodegenerative and cardiovascular diseases and several cancers [5,6]. Phenolic compounds not only have antioxidant effects, but also have strong antithrombotic, antimicrobial, antidiabetic, and antiatherogenic effects [124]. Besides, due to their scavenging activity, phenolic compounds play a crucial role in the stabilization of edible oils, protecting them against off-flavors development and enlarging their shelf life [97].

Total phenolic compounds (TPC) and total flavonoids are commonly determined by spectrophotometric techniques. TPC content is given as gallic acid equivalents (GAE), while total flavonoids are usually given as (+)-catechin equivalents. When analyzing single phenolic compounds, chromatographic methods are employed, such as HPLC-DAD/MS or LC-ESI-MS systems [52,125].

Oilseeds’ phenolic composition varies along with the species and, as mentioned above, depends on the extractive and refining techniques employed. Generally speaking, peanut contains proanthocyanidins and flavonoids as major constituents, and phenolic acids and stilbenoids as minor components. High quantities of these compounds are found in peanut skin, so avoiding pealing peanuts is beneficial for human health [126]. TPC in peanuts (82.64–92.12 mg GAE/g of seeds) is associated with favorable memory functions and inversely linked with stress response (cortisol, anxiety, and depression levels [127]. Soybeans are known to be rich in isoflavones and phenolic acids, with a TPC ranging from 5 to 25 mg GAE/g [125]. Certain phenolic compounds present in soybeans and flaxseed (e.g., hydroxytyrosol) show the peculiarity of forming complexes with proteins contained in these matrixes, facilitating their isolation [128]. In sesame seeds, TPC values are 146.25 mg GAE/g; and major phenolic compounds are phenolic acids (ferulic acid, gallic acid, protocatechuic acid and hydroxybenzoic acid), flavonols (quercetin) and certain lignans such as sesamin [129]. TPC in sunflower seed values are 22.29–33.09 mg GAE/g of seeds, mainly being phenolic acids, flavonoids and lignans [130]. In rapeseeds, the TPC is around 18 mg GAE/g, and the main constituents are sinapic acid derivatives and canolol [131]. Cottonseeds and safflower seeds show TPCs of 0.16 mg GAE/g (mainly, phenolic acids and isoflavones) [132], and 16 mg GAE/g (mainly, rutin and quercetin), respectively [133].

Stilbene derivatives, such as resveratrol, exert significant anti-inflammatory activity by inhibition of lipopolysaccharide induced nitric oxide (NO) production [134]. Stilbenoids are also reported to show cytotoxicity in vitro and in vivo in mouse macrophages and on human leukemia HL-60 cells; and neuroprotective effects [135]. Phenolic acids, such as sinapic acid, achieve scavenging and antiproliferative activities by reducing oxidative stress damage, which decreases ROS, pro-inflammatory cytokines (interleukin-6) and tumor necrosis factor-α (TNF-α) production. Flavonoids are great antioxidant agents and, although their antitumoral properties are not remarkable, they also produce anti-inflammatory effects by diminishing pro-inflammatory factors such as COX-2 expression, prostaglandin E2 and NO levels [136]. Proanthocyanins affect the lipidic metabolism, reducing FAs levels in rats, and playing an important role in hyperlipidemia, diabetes and obesity disorders [137]. Lignans are derived from two molecules of *p*-hydroxyphenylpropane, and usually act as monomers in the formation of lignins. Flaxseeds’ content of lignans is remarkable, with secoisolariciresinol diglucoside being the most abundant [138]. This phytochemical is a potent antioxidant, and behaves as precursor of phytoestrogens [139,140]. Besides, sesame seeds contain two significant groups of lignans: (1) oil-soluble lignans (sesamolin, sesamolinol, sesamin, pinoresinol, and sesaminol) and (2) glycosylated water-soluble lignin (pinoresinol triglucoside, pinoresinol monoglucoside, sesaminol triglucoside, and sesaminol monoglucoside) [141]. Consumption of lignans is associated with anticarcinogenic and hypolipidemic activities, also reducing atherosclerosis and CVDs’ incidence [142,143]. They also display immunomodulatory functions, neuroprotective function, antihypertensive activity, and prevent hypoxia and brain damage (Table 4).

Summarizing, phenolic compounds present in oilseeds represent great candidates for industrial applications, as their use is doubly advantageous: it improves oxidative stability and preservation in the commercial product, and exerts numerous beneficial and prophylactic effects in human health.

### 4.3. Phytic Acid

Phytic acid (inositol hexaphosphate (IP6)) is a bioactive phytochemical broadly distributed in plant foods (e.g., cereals, soybeans, rapeseeds, legumes, and enriched fiber foods). This compound is considered an anti-nutrient because when dissociated by ruminants’ digestive enzyme phytase, phytic acid (or its salt form, phytate) gives raise to inositol and phosphorus, two toxic molecules. However, for humans and non-ruminant animals phytates are not digestible, but can limit the bioavailability of nutrients such as minerals, proteins and starch [144]. Nevertheless, phytic acid possesses beneficial properties. Phytic acid shows antioxidant capacity by chelating iron ions, reducing site-specific DNA damage, and preventing tumor growth by suppressing the formation of the highly reactive OH• and other ROS. In animal studies phytic acid has been shown to inhibit neoplastic growth and metastasis in multiple types of cancer [145]. Phytates also show hypocholesterolemic capacity. The main oilseeds containing phytic acid are sunflower seed and rapeseed, with 1.52%, and sesame seed with 1.44–5.36%.

Analytical techniques to determine phytic acid in food matrixes include refractive index detection, gas chromatography (GC), and coupled plasma atomic emission spectrometry, although the most accurate is HPLC-UV [144]. Recent research has studied the elimination of this component from oilseeds using phytase, soaking, germination, fermentation, cooking, extrusion, dehulling, ultrasound waves, and high energy electromagnetic radiations [83,144].

### 4.4. Tocopherols

Tocopherols are oil-soluble antioxidant compounds present in numerous natural matrixes, such as oilseeds. They are a vitamin E complex component that shows four homologs: α (5,7,8-trimethyltocol), β (5,8-dimethyltocol), γ (7,8-dimethyltocol), and δ (8-methyltocol). Tocopherols act as biological free radical scavengers and may prevent infections, apart from its key nutritional properties [146]. The relative antioxidant activities in order of the homologs in vivo are α > β > γ  > δ, whereas a reversed order (δ > γ ≈ β > α) is observed when analyzed in oily systems in vitro. In vegetable oils, γ-tocopherol is the most abundant, and can prevent lipidic oxidation by scavenging lipid hydroperoxide radicals (LOO*). In comparison, *α*-tocopherol is essential for the diet and health effects of humans. Tocopherols constitute antitumor, antioxidant and hypocholesterolemic agents, diminishing the risk of heart diseases and some cancers. Thus, tocopherols are valuable nutraceuticals that offer health effects to consumers, while protecting oils from oxidative deterioration and rancidity. However, the extraction procedure can compromise the amount of tocopherols. Although each tocopherol shows different sensibility to degradation, in general terms, mild thermal treatments favor their release, while excessive intensity or temperature (over 180 °C) leads to decomposition [147].

Tocopherols are usually detected and quantified by chromatographic methods: GC, LC or HPLC [148,149,150].

### 4.5. Phytosterols

Phytosterols are plant-based compounds, present in many oilseeds. Phytosterols (sterols and stanols) are triterpenes of plants with disease preventive functions, particularly in cancers [7]. They also elicit anti-inflammatory, antioxidant, anti-tumoral, and antibacterial properties [147] (Table 4). Phytosterol-derived foods influence cholesterol absorption [151], increase its excretion, and decrease its gut absorption, which results in lower blood cholesterol levels [152]. Sesame seeds show the highest phytosterols content (400–413 mg/100 g). β-sitosterol, when compared with other phytosterols, was more thoroughly researched for its physiological effect and advantageous impact on human beings. β-Sitosterol reduces the levels of cholesterol, increases immunity, and displays anti-inflammatory effects [147].

Chromatographic techniques such as GC, LC, HPLC or even high temperature gas chromatography-mass spectrometry (HTGC-MS) are the most used methods for determining phytosterols, although spectrometries can also be performed with this purpose, as NIRS [153,154].

### 4.6. Dietary Fiber-Rich Foods

Flaxseed has been grown for fiber and also for food supplements for its possible health effects and medicinal uses [155] such as the prevention of cancer, heart related disease, obesity etc. [156,157]. Flaxseeds have been recently introduced with other food additives or nutraceuticals to increase the food nutritional quality [158,159]. Recent studies on rich-fiber functional foods and nutrients have led to a growing interest in flaxseed consumption, because of its higher content in omega-3FAs, fiber, phytoestrogens, and flavonoids among many involved in minimizing the effects of colorectal cancer (CRC) [62,111,160,161,162]. Flaxseed fiber greatly increased the fecal excretion of fat by 50% [110], reducing low-density lipoprotein (LDL) activity (Table 4).

### 4.7. Fatty Acids

Higher amounts of PUFAs enhance the oil quality for human intake. Flaxseeds contains 19.55 g omega-3 fatty acids per serving i.e., 85 g [124]. Dietary PUFAs exert anti-thrombotic, anti-inflammatory, hypolipidemic, anti-arrhythmic, and vasodilatory activities (oxylipins), and improve immunity [163,164]. In addition, the higher levels of linoleic acid decrease the systemic cholesterol levels, and play a crucial role in the prevention of atherosclerosis [115].

GC, UPLC-Q-Exactive Orbitrap mass spectrometry-based lipidomic method or HPLC-MS can be used to quantify these compounds [20,165].

The protective effect of high-oleic acid peanut oil and extra-virgin olive oil can be observed in rats with diet-induced metabolic syndrome by regulating branched-chain amino acids metabolism.

Alpha-Linolenic Acid (ALA) is an important omega-3 PUFA, which is necessary in the diet of humans. The daily intake of omega-3 PUFAs, such eicosapentaenoic acid (EPA) and docosahexaenoic acid (DHA), present in fish oils, is related with a lower risk of stroke [157], and colon cancer by changing the function and structure of membrane, raising the anti-inflammatory metabolites production and reducing cellular oxidative stress, out of other possible mechanisms [161]. Epidemiological studies reported the importance of daily consumption of ALA-derived flaxseed oil to reduce CRC (and many other cancer types) growth development [166,167,168,169] (Table 4). Inflammatory and oxidative stress markers were decreased by ALA in a model of inflammatory bowel disease [170]. Reactive oxygen species (ROS) play a pathogenic role in hypertension [171]. Interestingly, two studies evaluating the impact of enriched edible flaxseed oil in ALA have demonstrated a substantial reduction in Systolic Blood Pressure (SBP) (Table 4). A substantial inverse association between blood pressure and dietary ALA was observed throughout the INTERMAP study of a wide epidemiological trial of 4680 women and men [172]. In pre-hypertensive patients, 12 weeks of regular intake of 14 g of oil enhanced by 2.6 g ALA caused a reduction of 10 mm Hg and 3 mm Hg in diastolic blood pressure (DBP) and systolic blood pressure (SBP), respectively [114]. Flaxseed oil minimized DBP, SBP, and mean arterial blood pressure (MAP) by 10 mm Hg, 8 mm Hg, and 8 mm Hg, respectively, in patients with dyslipidemia who have ingested 15 mL flax oil including 8 g ALA/day in a meal, for 12 weeks [173]. A 4-week intake of ground flaxseeds decreased plasmatic pro-inflammatory oxylipins in the elderly [174].

### 4.8. Phytoestrogens

Great attention has been given to dietary phytoestrogens for their impact on human health. A number of other studies revealed the beneficial impact of phytoestrogens on human health, including on osteoporosis, cancers, menopausal symptoms, CVDs, obesity, T2D, antiproliferative, and male infertility [117,118]. A study reported that clinical symptoms of menopause can be decreased by consuming dietary phytoestrogens [118,175].

## 5. Future Perspectives

In the coming years, the development of new oilseed-based technology and products shall enhance human health benefits. The application of an oilseed mixture/blend in regular diet shall contribute to overcome chronic inflammatory state diseases such as cancers, CVDs, arthritis, diabetes, and obesity. Dietary lipids exert their effects both directly and indirectly by forming oxygenated metabolites with biologically active properties, such as eicosanoids and specific pro-resolving mediators. Because LC-PUFAs may compete for the same metabolic pathways, affecting the levels of bioactive metabolites in organs and tissues, it is of critical significance to assess the relative abundance of their precursors in cell membranes as a result of specific dietary habits [176,177].

Due to its medicinal properties, increasing oilseed meal intake is particularly beneficial, besides its nutritional values. Furthermore, the exploitation of oilseeds’ properties should continue to receive significant focus. Furthermore, food industrial applications of oilseed extracts—encapsulated or not—rich in phenolic compounds have demonstrated their remarkable value as natural additives to enhance products’ durability and organoleptic properties [126]. In addition, oilseeds can be used as feed for dairy animals, which leads to higher animal products’ supply and quality [178]. The growth of oilseed meal industries is incredibly significant, and with more people choosing to adopt vegetarian or vegan lifestyles, oilseeds are becoming part of our daily food, as they provide quality proteins, fats, and essential nutrients. Oilseeds, oilcake meals and edible oils demand is significantly increasing in the world with the continued development in per capital income, growing urbanization and population [179,180].

Regarding industrial no-food applications, oilseeds are being studied to produce biofuels, lubricants, biopolymers, surfactants, fibers or nanocarbons [19,181]. Additionally, the pharmaceutical industry could deeply investigate the optimization of the administration of the bioactive compounds found in oilseeds to improve their therapeutic action and assure their bioavailability, to profit from their valuable functional effects. In addition, for industrial and medical purposes, certain enzymes can be produced using oilseeds as a fermentation substrate [22].

Besides, oil production waste could be used as a raw material to obtain valuable compounds: a wide range of phenolic compounds (proanthocyanidins, flavonoids, phenolic acids, and stilbene derivatives), can be extracted from peanut skin [126]; tocopherols and phytosterols recovery from rapeseed oil production waste [50]; bioethanol could be produced from peanut by-products, etc. These compounds are easily and conveniently applicable in the food and pharmacy industries, as excipients, as preservation agents and/or as biofunctional molecules [126]. In this way, a circular economy could be promoted. Oilseed byproducts could fit in the industrial, cosmetic, and medicinal fields. Comprehensive studies should be developed to explore more of their applications in the future [19].

## 6. Conclusions

The types of oilseeds, the health benefits of oilseeds, the various types of bioactive compounds and extraction methods of oilseeds have been reviewed in this article. It mainly focuses on human health and the role of different oilseeds and their bioactive compounds in a healthy and balanced diet. The consumption of Long Chain-PUFAs, MUFAs, and polyphenols from edible oils has been linked to lower levels of oxidative stress and inflammation, according to available evidence. Future research on this topic promotes the enhanced use of oilseeds as bioactive, functional compounds and healthy foods. In this review article some of the methods used for the extraction of vegetable oils from oilseeds, which can increase the oil yield and enhancing oil quality, are described. Conventional methods had more disadvantages due to the long extraction times required, the consumption of large amounts of organic solvents and the high temperatures employed which can have adverse effects and deteriorate the quality of the extracted oil. Nevertheless, new and innovative techniques have been developed allowing the effective extraction of oilseeds while preserving its properties. Additionally, oilseed crops and oilseed meal were found to be a good protein and minerals source. They can be added in food products to fight malnutrition in the world or to treat different chronic inflammatory-state diseases, including those that might be caused (at least in part) by TFAs. Besides, oilseed meals can be used as feed for livestock, which can contribute to improving their health and stimulate milk production.

## Figures and Tables

**Table 1:** Production rate of different oilseeds in India. Data are expressed as: Area (×10^3^ hectares)/Production (×10^3^ metric tons). Sources: USDA Official and Ministry of Agriculture, Government of India.

Oilseed	2016–2017	2017–2018	2018–2019	2019–2020
Peanut	-	5000/6650	4850/4720	4900/6300
Flaxseed	325.2/184.4	326.2/173.9	172.7/99.1	-
Rapeseed	-	6600/6450	7200/8000	7400/7700
Sunflower	-	330/230	270/172	250/182
Sesame	1666.9/746.8	1579.8/755.1	1420/650.4	-
Soybean	-	10,550/8350	11,500/10,930	12,000/9300
Cottonseed	-	12,450/12,312	12,600/10,953	13,300/12,949
Safflower	144.3/93.9	82.2/55.3	45.9/24.7	-

**Table 2:** Nutrient content of oilseeds (100 g) (adapted from Food Standards Agency https://www.food.gov.uk/ (accessed on 16 October 2020), and Food Research Institute https://www.foodresearchgh.org/ (accessed on 16 October 2020), (2002)).

Nutritional Composition	Flaxseed	Rapeseed/ Canola	Sunflower	Groundnut	Sesame/ Benne Seed	Safflower	Cotton Seed	Soybean
Energy (Kcals) **	534.0	884.0	163.0	570.0	573.0	517.0	367.0	173.0
Proteins (g)	19.5 ^#^	22.0 *	19.8	25.6	18.2	16.2	32.6 ^#^	14.0
Carbohydrates (g)	34.3 ^#^	8.3 *	18.6	12.5	0.9	34.3	21.9 ^#^	5.1
Fiber(g)	27.9 ^#^	7.2 *	6.0	6.2	7.9	N	5.5 ^#^	6.1
Fat(g)	34.0 ^#^	9.6 **	47.5	46.0	58.0	38.5	36.3 ^#^	7.3

^#^ Data from [1], * data from [14]; ** data from www.nutritionvalue.org/seeds (accessed on 16 October 2020); N, precise quantity information is not available.

**Table 3:** Mineral content of oilseeds (100 g) (adapted from Food Standards Agency https://www.food.gov.uk/ (accessed on 16 October 2020), and Food Research Institute https://www.foodresearchgh.org/ (accessed on 16 October 2020).

Macro/Micro Nutrients (mg)	Flaxseed *	Rapeseed **	Sunflower	Peanut	Sesame	Safflower	Cotton *	Soybeans
Calcium	199.0	400.0	110.0	60.0	670.0	78.0	100.0	83.0
Phosphorus	498.0	800.0	640.0	430	720.0	644.0	800.0	250.0
Potassium	681.0	800.0	710.0	670	570.0	687.0	1350.0	510.0
Sodium	34.0	5.0	3.0	2.0	20.0	3.0.0	25.0	1.0
Zinc	4.2	N	5.1	3.5	5.3	5.1	6.0	0.9
Magnesium	362	250.0	390	210.0	370.0	353.0	440.0	63.0
Iron	6.2	N	6.4	2.5	10.4	4.9	5.4	3.0

Data from [1]; * data from [14]; ** data from www.nutritionvalue.org/seeds (accessed on 16 October 2020); N, precise quantity information is not available.

**Table 4:** Bioactive compounds and their health beneficial effects.

Bioactive Compounds	Beneficial Effects	References
Glucosinolates	Preventing carcinogenesis, decrease cancer incidence at stomach, rectum, colon, and lung.	[91]
Phenolic compounds	Antioxidant, antimicrobial, antiviral, antihypertensive, anti-inflammatory, immunomodulatory and anticancer activities; neurodegenerative and cardiovascular diseases prevention; protection against UV radiation.	[95,96,97,98,99,100]
Phytic acid	Hypocholesterolemic, anticancerous activities, inhibits the metastasis of tumor.	[101,102,103]
Phytosterols	Anti-inflammatory, antioxidant, antibacterial properties	[104,105,106]
Tocopherols	Preventing cancer and heart diseases, antioxidative activity and nutritional values.	[107,108]
Dietary Fiber	Minimize heart disease risk, colorectal cancer, inflammation, diabetes and obesity, cholesterol lowering activity, increased the fecal excretion of fat.	[109,110]
Alpha-linolenic acid	Decreased tumor growth, reduction in (SBP), lower growth rate of breast and colon cancers, reduction of (DBP, SBP).	[111,112,113,114]
PUFA	Prevention of atherosclerosis	[115]
Phytoestrogens	Osteoporosis, cancer, menopausal symptoms, cardiovascular disease, obesity and type 2 diabetes, male infertility	[116,117,118]

Regarding the analytical techniques employed to determine glucosinolates in vegetable samples, most of them are liquid chromatography (LC) coupled to different detectors, such as LC-MS, LC-MS/MS, HPLC-DAD-MS or UHPLC-Q-Orbitrap-MS [119,120]; although Vis-NIRS spectroscopy has recently been proven to also be effective [121].

## Data Availability

Not applicable. All Data is contained within the article and all Data presented in this work are available in a publicly accessible repository which are clearly mentioned within this manuscript.
